# Fabrication and photoresponse of ZnO nanowires/CuO coaxial heterojunction

**DOI:** 10.1186/1556-276X-8-387

**Published:** 2013-09-17

**Authors:** Jen-Kai Wu, Wei-Jen Chen, Yuan Huei Chang, Yang Fang Chen, Da-Ren Hang, Chi-Te Liang, Jing-Yu Lu

**Affiliations:** 1 Department of Physics, National Taiwan University, Taipei 106, Taiwan; 2 Graduate Institute of Applied Physics, National Taiwan University, Taipei 106, Taiwan; 3 Department of Materials and Optoelectronic Science, National Sun Yat-sen University, Kaohsiung 804, Taiwan; 4 Center for Nanoscience and Nanotechnology, National Sun Yat-sen University, Kaohsiung 804, Taiwan; 5 School of Electronic and Electrical Engineering, Sungkyunkwan University, Suwon 440-746, South Korea

**Keywords:** ZnO nanowires, CuO, Coaxial heterojunction

## Abstract

The fabrication and properties of *n*-ZnO nanowires/*p*-CuO coaxial heterojunction (CH) with a photoresist (PR) blocking layer are reported. In our study, *c*-plane wurtzite ZnO nanowires were grown by aqueous chemical method, and monoclinic CuO (111) was then coated on the ZnO nanowires by electrochemical deposition to form CH. To improve the device performance, a PR layer was inserted between the ZnO buffer layer and the CuO film to serve as a blocking layer to block the leakage current. Structural investigations of the CH indicate that the sample has good crystalline quality. It was found that our refined structure possesses a better rectifying ratio and smaller reverse leakage current. As there is a large on/off ratio between light on and off and the major light response is centered at around 424 nm, the experimental results suggest that the PR-inserted ZnO/CuO CH can be used as a good narrow-band blue light detector.

## Background

Because of its wide band gap (3.37 eV) and large exciton binding energy (60 meV), zinc oxide (ZnO) is one of the most promising materials for optoelectronic device applications in the ultraviolet (UV) region [1-3]. ZnO thin films can be produced by several techniques, such as reactive evaporation, molecular beam epitaxy (MBE) [4-6], magnetron sputtering technique [7], pulsed laser deposition (PLD) [8], sol–gel technique [9], chemical vapor deposition, electrochemical deposition [10], and spray pyrolysis [11]. In recent years, ZnO-based heterojunctions have been extensively studied for application as UV photodetectors. These ZnO-based heterojunctions can be classified into two categories: thin film heterojunction (FH) and coaxial heterojunction (CH). ZnO/SiC [2], ZnO/NiO [12], and ZnO/GaN [13] belong to the category of thin film heterojunction which had been shown to possess good photoresponse in the UV region. On the other hand, *p*-copper oxide (CuO)/*n*-ZnO nanowires (NWs) [14], which belong to the category of coaxial heterojunction, were found to have large enhancement in photocurrent under UV illumination.

ZnO NW possesses many attractive advantages over ZnO thin film. The light trapping ability and great photosensitivity owing to the presence of an oxygen-related hole-trap state at the ZnO NW surface [15] make ZnO NW-based heterojunction very attractive for use as a photodetector. Due to the good optical properties of ZnO NWs and the strong absorption of CuO in the visible region [16], ZnO NW/CuO heterojunction has drawn much interest these days. A wide variety of processes, including sputtering method [14], sol–gel technique [17], thermal oxidation [18], and modified hydrothermal method [19], have been developed to fabricate ZnO/CuO CH. These works demonstrated that good rectification ratio and good photoresponse can be obtained with ZnO/CuO coaxial heterojunctions. However, in coating a CuO layer on ZnO nanowires, it is unavoidable that part of the CuO will be in contact with the ZnO buffer layer, and as there are two parallel channels for current conduction (one from the ZnO buffer layer to the CuO layer, and the other from ZnO nanowires to the CuO layer), it is not possible to take full advantage of the benefits that are associated with using the ZnO nanowires in making the photodetector [14,18,19]. In this letter, we report fabrication and characterization of a ZnO nanowire/CuO heterojunction photodetector with a photoresist blocking layer. In our study, the ZnO NWs were grown by hydrothermal method, and the sample was then spin-coated with a photoresist layer before the growth of the CuO layer. Structural investigations of the coaxial heterojunction indicate that the sample has good crystalline quality. It was found that our refined structure possesses a better rectifying ratio and a smaller reverse leakage current which are 110 and 12.6 μA, respectively. With the increase of reverse bias from 1 to 3 V, the responsivity increases from 0.4 to 3.5 A W^−1^ under a 424-nm light illumination.

## Methods

ZnO NW arrays were grown on an indium tin oxide (ITO)-coated glass substrate by aqueous chemical method as reported in [20]. The reaction solution was 0.05 M Zn(NO_3_)_2_ · 6H_2_O mixed with 0.05 M C_6_H_12_N_4_. The growth temperature and time are 90°C and 2 h, respectively. After the growth, the sample was baked at 100°C for complete dryness. In order to provide electrical blocking between the ZnO buffer layer and the CuO film, a layer of photoresist (DSAM) was spin-coated on ZnO NW arrays as a blocking layer. To remove the PR on top of the ZnO NWs, acetone was dropped onto the sample while it is spinning in a spin coater. With this method, the upper part of the nanowires is not covered by the PR but the bottom part of the nanowires and the ZnO buffer layer are still coated with PR, thus ensuring that the CuO layer which will be grown later will not be in contact with the ZnO buffer layer. Copper was then coated on ZnO NWs by ECD and was then annealed at 400°C for 2 h with the oxygen flow offset at 20 sccm [17]. Finally, a 100-nm silver layer was deposited onto the CuO layer by thermal evaporation to serve as an ohmic contact for electrical measurements. The morphology of ZnO/CuO was examined using a HITACHI S-2400 scanning electron miscroscope (SEM; Chiyoda-ku, Japan). The crystal structure was examined using a transmission electron microscope (TEM; Philips Tecnai G2 F20 FEG-TEM) located at the Department of Physics, National Taiwan University, and by X-ray diffraction (PANalytical X’Pert PRO, Almelo, The Netherlands). Optical transmission spectra were measured using a JASCO V-570 UV/VIS/NIR spectrophotometer (Easton, MD, USA). Xenon arc lamp (LHX150 08002, Glasgow, UK) and iHR-320 monochromator (HORIBA Scientific, Albany, NY, USA ) were used in the photoresponse measurement, and the current–voltage (*I-V*) curves were measured using Keithley 236 and 4200-SCS (Cleveland, OH, USA).

## Results and discussion

**Figure 1:**
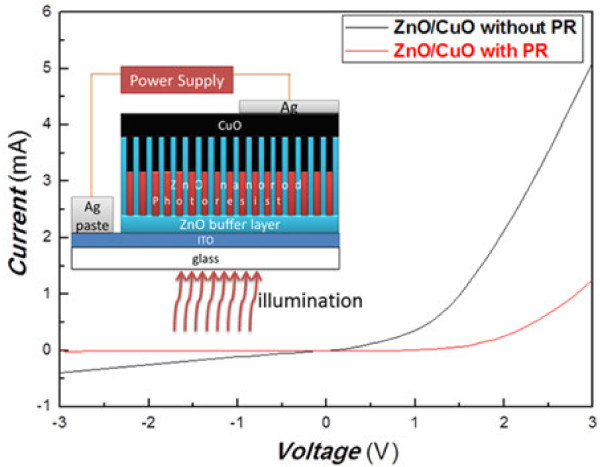
***I*-*V *characteristic curves of ZnO/CuO without PR (black line) and ZnO/CuO CH with PR (red line).** The inset shows a schematic diagram of the sample structure with PR as an insulating layer.

The inset in Figure 1 shows the schematic of the sample structure and the measurement setup for the *I*-*V* measurement of the ZnO-CuO heterojunction. Figure 1 depicts the *I*-*V* curves of the ZnO/CuO heterojunction without PR and with PR as an insulating layer. We can see quite clearly in this figure that both devices have a characteristic *p*-*n* junction rectifying behavior. We can notice in this figure that the reverse bias leakage current in the ZnO/CuO heterojunction without PR is quite large (0.45 mA at −3 V). On the other hand, the leakage current is greatly suppressed for the sample with PR inserted in ZnO/CuO CH. In addition, we also find that at a bias of 3 V, the rectifying ratio of the former and the latter is 8 and 110, respectively. Thus, the ZnO/CuO CH with PR shows a better rectifying ratio compared with the ZnO/CuO heterojunction without PR. The results demonstrate clearly that adding a PR blocking layer can reduce the reverse leakage current and improve the rectifying ratio.

**Figure 2:**
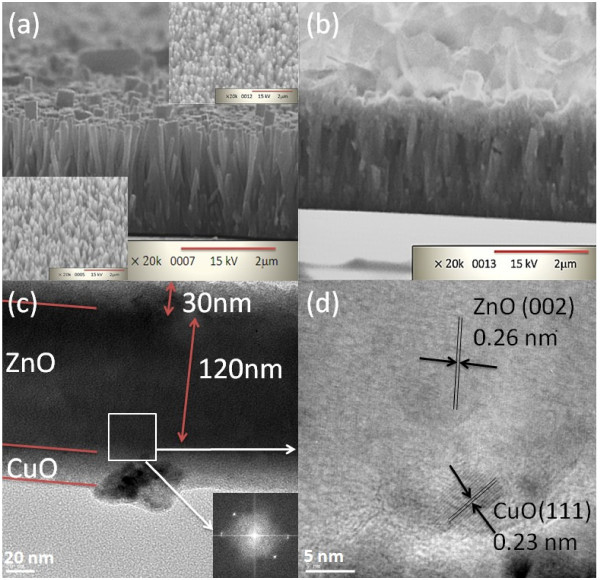
**SEM and TEM images and FFT.** SEM images of the cross-sectional view of **(a)** ZnO NW arrays and **(b)** ZnO NWs/CuO CH. Bottom-left and top-right insets in **(a)** show tilt views of ZnO NWs and PR on ZnO NWs, respectively. **(c)** Low-magnification TEM image and FFT (inset) of ZnO/CuO CH. **(d)** High-magnification TEM image of the ZnO/CuO interface taken from the square region drawn in Figure 2**c**.

Figure 2a shows SEM images of the cross-sectional view of ZnO NW arrays. We can see in this figure that ZnO NWs were grown perpendicularly to the ITO substrate. The bottom-left inset in this figure is the image of the tilt view of ZnO NW arrays, and the top-right inset is the image of the ZnO NWs with PR on top being removed by acetone. We note from the top-right inset that about 200-nm-long ZnO on top of ZnO NWs was not covered by PR. Figure 2b is the image taken after the CuO layer was deposited. For TEM measurement, the sample was put in absolute alcohol and was then vibrated ultrasonically. Subsequently, the solution was dropped onto copper grids with carbon film. The TEM image of ZnO/CuO CH shown in Figure 2c indicates that the diameter of the ZnO NW and the thickness of the CuO layer are about 120 and 30 nm, respectively. A fast Fourier transform (FFT) pattern obtained from the square region marked in Figure 2c indicates two lattice planes. The FFT analysis shows that the *d*-spacing calculated from the electron diffraction spots are estimated to be around 0.26 and 0.23 nm. Figure 2d shows two groups of parallel fringes with the *d*-spacing of 0.26 and 0.23 nm which correspond to the (002) plane of wurtzite ZnO and the (111) plane of monoclinic CuO, respectively.

**Figure 3:**
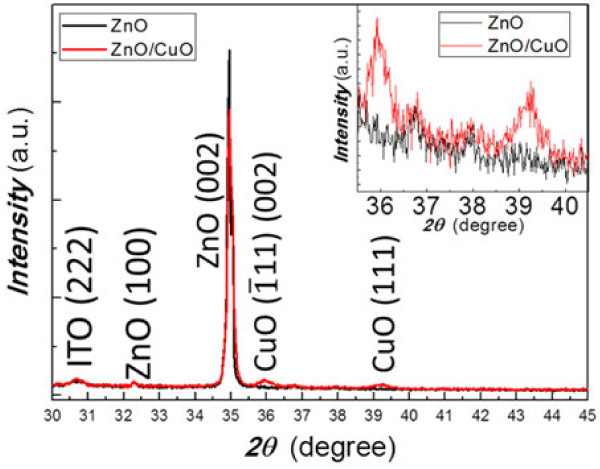
**XRD patterns of ZnO (black line) and ZnO/CuO (red line).** The inset shows the XRD patterns of ZnO (black line) and ZnO/CuO (red line) between 2*θ* = 35.5° and 40.5°.

The XRD patterns of ZnO NWs and ZnO/CuO CH are shown in Figure 3. For the ZnO NWs, the peaks at 30.5°, 32.3°, and 34.9° are the diffraction peaks from ITO (222), ZnO (100), and ZnO (002), respectively. Two extra peaks at 35.8° and 39.2° show up for the ZnO/CuO CH structure, corresponding to the diffraction from CuO $\left(\bar{1}11\right)$ and CuO (111), respectively [16-18]. The weak intensities of two CuO peaks indicate that the CuO film is very thin, and it can be seen that there is a small shift of the XRD patterns, which is possibly due to the strain generated by the lattice mismatch. We did not find any peak that corresponds to the diffraction from Cu_2_O (111) or Cu (111) which would be located at 36.4° and 43.3°, respectively [18]. The XRD results are consistent with the TEM results that a pure CuO has been grown successfully on top of ZnO NWs.

**Figure 4:**
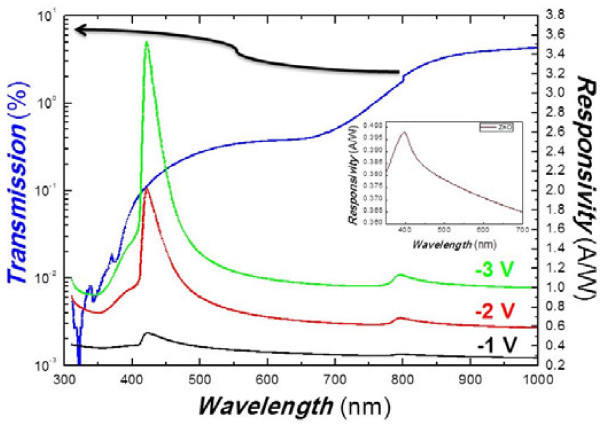
**Transmission spectrum of ZnO/CuO CH and its photoresponse spectrum at different reverse biases.** The inset shows the photoresponse of ZnO NWs for comparison.

Transmission and spectral photoresponse of the ZnO-CuO are shown in Figure 4. With the light coming from the ‘back’ of the sample as shown in the inset of Figure 1, the ITO/glass substrate acts as a ‘low-pass filter’ and will allow the light with a wavelength above 350 nm to pass without absorption [21]. As can be seen in the figure, the transmission spectrum of ZnO/CuO CH (blue line) shows two abrupt drops, one at about 420 nm and the other at about 800 nm, which correspond to the band-edge absorption of ZnO and CuO, respectively. Also shown in the figure are the photoresponse spectra of ZnO/CuO CH under different reverse biases. We can identify two features located at 424 and 800 nm in the spectra. The huge response around 424 nm is below the typical band gap of ZnO. It could be due to the narrowing of the band gap of ZnO as a result of tensile stress in the coaxial structure [22], which is consistent with our XRD and TEM results. Another response around 800 nm can be attributed to the photoresponse of CuO [23]. It is much smaller than that of the main peak at 424 nm because the CuO film is thin. We note that the optical responsivity of the devices is bias sensitive. The responsivity of the sample at 424 nm increases from 0.4 to 3.5 A W^−1^ when the reverse bias increases from 1 to 3 V.

**Figure 5:**
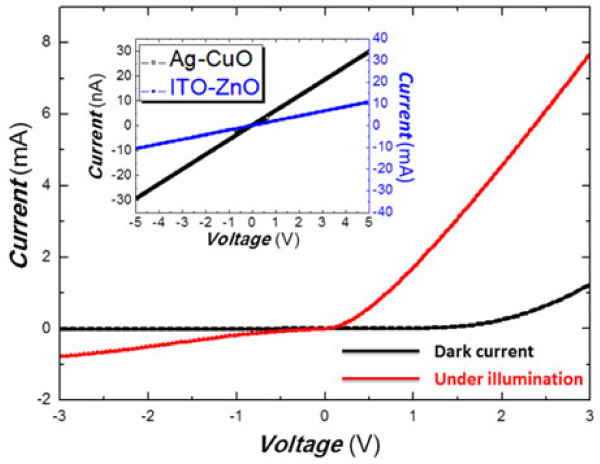
***I*-*V *characteristic curves of the ZnO/CuO CH with PR.** In the dark (black line) and under light (424 nm) illumination (red line). The inset shows the *I-V* curves of the Ag-CuO film (black line) and ITO-ZnO NWs (blue line).

The *I-V* curves of PR-inserted ZnO NWs/CuO with and without light illumination are shown in Figure 5. The inset shows that the *I*-*V* curves for the Ag-CuO film (black line) and ITO-ZnO NWs (blue line) are both linear, indicating the contacts are ohmic [24-26]. Hence, the characteristic rectifying behavior is due to the ZnO/CuO CH *p-n* junction [26]. As can be seen in the figure, the leakage current is 12.6 μA at a reverse bias of −3 V, and it increases to 770 μA under light illumination, which is an increase of about 60-fold. As there is a large on/off ratio and the photoresponse is centered at around 424 nm, the experimental results suggest that the PR-inserted ZnO/CuO CH can be used as a good narrow-band blue light detector [27].

## Conclusions

In summary, PR-inserted ZnO nanowires/CuO coaxial heterojunctions were fabricated by a low-cost and simple method. Structural studies demonstrated that the nanostructure has good crystalline quality. Optical and electrical characteristics were studied by transmission spectrum, current–voltage curve, and photoresponse measurements, and it is found that adding a PR blocking layer can effectively reduce the reverse bias leakage current and enhance the rectifying ratio. For our sample, the turn-on voltage is 1.7 V, the rectifying ratio between 3 and −3 V is 110, and the responsivity is 3.5 A W^−1^ at a reverse bias of 3 V in the visible region. As there is a large on/off ratio between light on and off and the light response is centered at around 424 nm, the experimental results suggest that the PR-inserted ZnO/CuO CH can be used as a good narrow-band blue light detector.

## Competing interests

The authors declare that they have no competing interests.

## Authors’ contributions

JKW and WJC performed the experiments and fabricated the devices. YHC and YFC coordinated the project. JYL performed the TEM measurements. JKW, DRH, and CTL drafted the paper. All authors read and approved the final manuscript.
